# Sudden Cardiac Death in Athletes - What Can be Done?

**Published:** 2006-07-01

**Authors:** Joydeep Ghosh

**Affiliations:** Assistant Professor of Medicine, Maimonides Medical Center, 4802 Tenth Avenue, Brooklyn, NY 11219, USA

**Keywords:** sudden cardiac death, exercise, athletes

## Abstract

Sudden death in athletes is a rare event but brings with it an impact that goes beyond sport. There are many causes of sudden death during exercise. While the responsibility of preventing or treating them lays with us physicians, preparticipation screening is largely ineffective and impractical. Definitive, large scale prospective research is required in order to design the most cost-effective system for screening of athletes. In the meanwhile rapid access to defibrillators by trained personnel remains the best possible approach to abort sudden death.

With the World Cup in soccer - arguably the most passionate sporting event of our times - only a few days away, it is appropriate to look at the lessons we have learned over the past few years about sudden death in competitive sport.

In 490 BC Phidippides ran 26.2 miles from Marathon to Athens, delivering the news of the Greek victory over the Persians (the first 'marathon' in recorded history), but then immediately collapsed and died. This is probably the first recorded incident of sudden death of an athlete. More recently  the death of some high-profile athletes have focused public interest on this infrequent event and current data reveal that in the United Sates alone, about 100 athletes - ranging from schoolchildren to professionals - die every year while engaged in sporting activity [1]. In India accurate data are not available but recently media attention was focused on a Brazilian soccer player who died while playing for his adopted club. Across societies around the world athletes are considered to be the pinnacle of health and given high status; death of a young, well-trained athlete on the field represents a particularly tragic event that leads to utter dismay within the family and the community and often a barrage of negative media attention.

The paradox of exercise is that while in the long-term it generally attenuates the risk of sudden cardiac death, the relative risk is actually increased during the time of exercise and up to 30 minutes following it [2]. This is especially so in patients with hypertrophic cardiomyopathy, congenital anomalous coronary artery abnormalities, and inherited ion channelopathies like long QT syndrome and catecholaminergic polymorphic ventricular tachycardia, or Arrhythmogenic Right Ventricular Cardiomyopathy, and it is no surprise that patients with these conditions have a risk of sudden death with sport that is well above normal. Other conditions implicated in sudden death in athletes include accessory pathways with antegrade conduction properties, bicuspid aortic valve, myocarditis, ruptured aortic aneurysm, Marfan syndrome, mitral and tricuspid valve prolapse and ingestion of stimulants like cocaine and anabolic steroids. In addition, sudden death on the field can also be caused by certain activities like trauma to the chest (commotio cordis) or to the neck (causing asystole from carotid sinus reactivity) [3].

Given the myriad causes listed above, the question we have to answer is whether pre-participation screening works. Since the early eighties, Italy has been in the forefront of efforts to identify athletes perceived to be at risk and withdraw them from competitive sports, with a preparticipation screening program based on 12-lead ECG, a family and personal history and physical examination [4]. Data presented recently [5] reveal a striking decline in mortality in the population studied (12-35 year olds, in the Veneto region) with the incidence of sudden death declining to one-tenth of what it was at the beginning of the study. Most of this was attributed to fewer cases of athletes dying from cardiomyopathies that were detectable on ECG. To the best of my knowledge, there are no other prospective studies (of any magnitude) that look at the effect of preparticipation screening in athletes.

Whether the result of this study is applicable to other countries is of course, questionable. Northern Italy has an unusually high incidence of ARVD in its homogenous population, and unless one is dealing with similar circumstances the results may not be reproducible. In the United States currently there is no advocacy for routine ECG's, and the diagnostic tool used most frequently is a detailed history and physical examination, with particular attention paid to syncope, palpitations, chest pain or sudden death in the family. ECG, stress testing and echocardiograms are used only if there is a suspicion of high risk from the history elicited. Interpretations of 'quick-screen' echocardiograms, while being effective in diagnosing HCM or ARVC in a short period of time, may be particularly challenging given the similarities between "athletes heart" and hypertrophic cardiomyopathy.

With regard to India and other developing countries, such preparticipation screening will need an enormous increase in the Government's commitment to public health given the huge population of people that needs to be screened and the low incidence of disease that cause sudden death (a notable exception may be hypertrophic cardiomyopathy, which has been reported to have an incidence of 1 in 500 in the Western population). Unfortunately I doubt that this 'high investment, low return' policy will find many takers in a country like India, though saving even one young adult or child's life ought to be seen as making an investment in the future. The other significant problem with screening is identifying false positives, with the unnecessary psychological burden it places on athletes and their families [6]. If any kind of screening policy is indeed adopted, we need physicians who are well-trained to identify the diseases they are seeking, and who know how to proceed with borderline cases without causing unnecessary alarm.

If preparticipation screening is far away in a country like India, the least we can do is provide external defibrillators in as many athletic events as possible, and train paramedics to use them properly. In particular they must be able to recognize cardiac arrest quickly, and deliver defibrillation promptly. A successful outcome depends primarily on time to defibrillation, with about a 10% per-minute decrease in survival [5].

In the meanwhile, enjoy the World Cup. Strict compliance to FIFA guidelines at all venues in Germany regarding availability of rapid access defibrillation will hopefully ensure that no gifted athlete dies suddenly on the field.
